# Utilizing Design of Experiments Approach to Assess Kinetic Parameters for a Mn Homogeneous Hydrogenation Catalyst

**DOI:** 10.1002/cctc.202101140

**Published:** 2021-09-14

**Authors:** Robin K. A. van Schendel, Wenjun Yang, Evgeny A. Uslamin, Evgeny A. Pidko

**Affiliations:** ^1^ Inorganic Systems Engineering Department of Chemical Engineering Delft University of Technology Van der Maasweg 9 2629 HZ Delft (The Netherlands

**Keywords:** Catalytic hydrogenation, Design of experiments, High-throughput experimentation, Reaction kinetics, Statistical analysis

## Abstract

Homogeneous hydrogenation catalysts based on metal complexes provide a diverse and highly tunable tool for the fine chemical industry. To fully unleash their potential, fast and effective methods for the evaluation of catalytic properties are needed. In turn, this requires changes in the experimental approaches to test and evaluate the performance of the catalytic processes. Design of experiment combined with statistical analysis can enable time‐ and resource‐efficient experimentation. In this work, we employ a set of different statistical models to obtain the detailed kinetic description of a highly active homogeneous Mn (I) ketone hydrogenation catalyst as a representative model system. The reaction kinetics were analyzed using a full second order polynomial regression model, two models with eliminated parameters and finally a model which implements “chemical logic”. The coefficients obtained are compared with the corresponding high‐quality kinetic parameters acquired using conventional kinetic experiments. We demonstrate that various kinetic effects can be well captured using different statistical models, providing important insights into the reaction kinetics and mechanism of a complex catalytic reaction.

## Graphical abstract

**Statistical modelling**: A rapid and powerful approach to assess the catalytic reaction kinetics is developed by combining the design of experiments with statistical modelling. Equating the regression equations and derived coefficients of resulting statistical models, we are able to measure the activation energy, to capture different reaction regimes, and to map the response of the observed reaction rate to condition parameters.

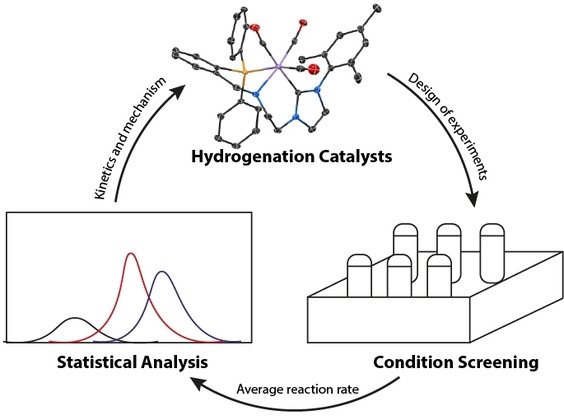

## Introduction

Rapidly increasing demand in chemicals and fuels puts forward new challenges for chemical industry and chemistry in general. The fast implementation of novel catalytic processes is therefore becoming crucial as it enables more sustainable and effective chemical transformations.^[1]^ The transition from laboratory‐scale catalytic research to industrial application is often a limiting step. This requires deep understanding of the behavior of the system on both molecular and reactor scale. Implementing data‐driven approaches can help solve this complex problem by providing descriptive non‐biased models.^[2]^ Design of experiment and statistical analysis of the experimental data are key ingredients within this approach.^[3]^

**Figure 1:**
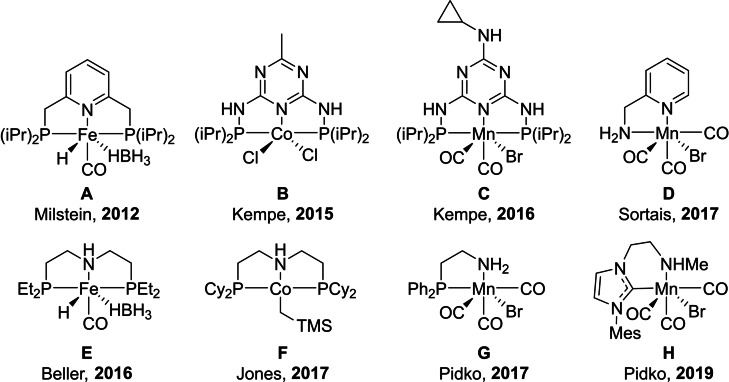
Selected examples of 3d metal complexes active in hydrogenation reactions.[^8^, ^9^, ^10^, ^11^, ^12^, ^13^, ^14^, ^15^]

Reduction of carbonyl compounds is an important process widely applied in the fine and bulk chemical industry. In contrast to conventional stoichiometric reactions yielding vast amounts of inorganic waste, catalytic hydrogenation utilizing molecular hydrogen represents an environmentally‐friendly and atom‐efficient alternative.^[4]^ This becomes especially attractive as the hydrogen market has been rapidly expanding over the last few years. Hydrogenation reactions require the use of a catalyst. Over the last decades, a number of highly active homogeneous carbonyl hydrogenation catalysts based on defined transition metal complexes, e. g. ruthenium, iridium, and rhodium, were developed. Many of these catalysts enable highly selective homogeneous hydrogenation under mild conditions.[^4b^, ^5^] However, the possibility to use cheaper earth‐abundant 3d metal complexes as an alternative to their noble metal based counterparts has drawn considerable attention recently.[^6^, ^7^] Figure 1 presents selected representative examples of such highly‐active catalysts. Fe‐**A**,^[8]^ Co**‐B**,^[9]^ and Mn‐**C** ^[10]^ containing lutidine‐ and diamino triazine‐derived pincer ligands were found to be highly active in the hydrogenation of ketones and aldehydes and can operate at 0.05–0.25 mol. % catalyst loading. Amino ligand‐based complexes (**E**,^[11]^ **F**,^[12]^ and **G** ^[13]^) represent the most potent 3d metal catalysts for ester hydrogenations requiring 0.2–2 mol. % catalyst loading. Mn‐based complexes (e. g. **D^[^** ^14**]**^, **H** ^[15]^) have emerged as highly efficient transfer hydrogenation catalysts enabling excellent performance at catalyst concentrations as low as 75 ppm.

Modern synthetic protocols allow obtaining well‐defined catalytic complexes with nearly any given ligand environment. In contrast, the testing procedures used to evaluate the catalyst performance are less defined. The limited scope of conditions used during the initial catalyst screening phase often result in a situation when important effects including the catalyst activation, its stability under reaction conditions and different deactivation pathways are overlooked. This may lead to a limited understanding of the intrinsic catalyst activity and therefore hamper the establishment of solid predictive structure‐performance relationships. The availability of kinetic data and its accurate modelling would provide a comprehensive insight in the behavior and mechanisms of catalytic reactions and therefore facilitate further development and optimization of highly efficient hydrogenation catalyst systems.^[16]^

**Figure 2:**
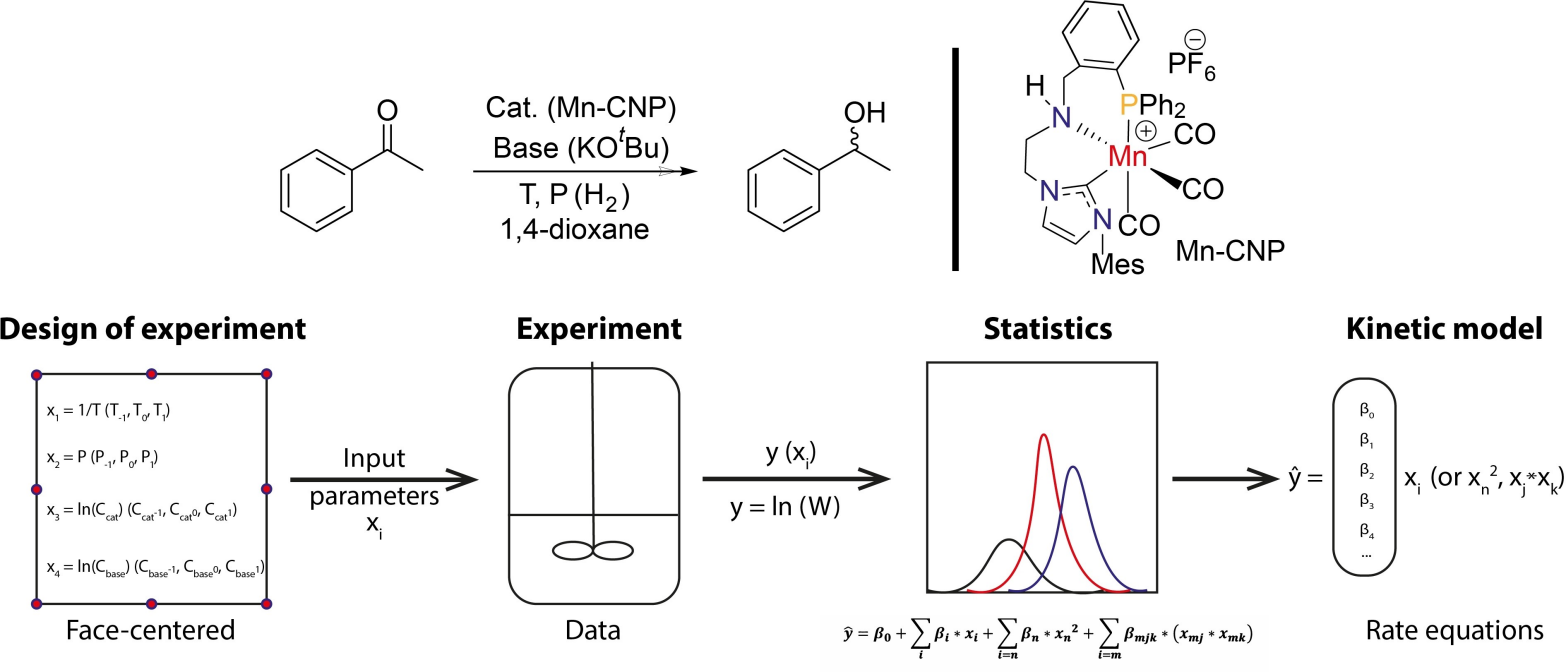
Reaction schematics showing the hydrogenation of acetophenone in presence of MnCNP catalyst (top); the schematics of the experimental workflow (bottom).

The modelling of homogeneous catalytic reactions can be based on different approaches ranging from a purely empirical description to formal kinetics to theory‐assisted micro kinetic modelling.^[17]^ The resulting rate equations can therefore take various forms ranging from simple power laws to highly complex polynomial forms. Due to the complexity of the catalytic networks, these models may not fully describe the system in many cases. In the specific case of hydrogenation catalysis, the situation is further complicated by the experimental challenges related to the use of high‐pressure equipment. Thus, the development of precise and rapid kinetic modelling approaches based on minimum experimental runs become indispensable. Herein we apply the response surface Box‐Wilson statistical methodology^[18]^ to the kinetic analysis of ketone hydrogenation reaction catalyzed by a Mn (I) pincer complex (Figure 2) recently reported by us as a highly active ketone hydrogenation catalyst.^[19]^ Containing N‐heterocyclic carbene, phosphine and nitrogen donor centers, we will refer to it as Mn‐CNP. Response Surface Design (RSD) is shown to be efficient as a tool to provide a rapid access to the “classic” kinetic parameters with less experimentation. Furthermore, we demonstrate that this approach can also indicate hidden parameters which are not observed during the conventional kinetic experiments.

## Results and Discussion

### Design of Experiment

The experimental setup was designed according to the Box‐Wilson methodology^[18]^, specifically a response surface design using the central composite face‐centered type. Four continuous regressors in three levels were chosen (temperature, H_2_ pressure, concentration of the catalyst and concentration of the base) and kept un‐coded. Due to uncertainty in measuring initial reaction rates, the chosen regress and was the average reaction rate (measured as product*t ^−1^). The average rate was calculated as the concentration of produced alcohol divided by the reaction time in hours. No blocks were used and all runs were randomized save for the temperature due to the home‐built autoclave heating six reactions at a time in unison. With all the cube points, axial points and replicates, a total of 30 runs were performed. The reaction loadings and conditions are summarized in *Table S1*.

### Response Surface Design

A central composite face‐centered design was used to obtain a multiple polynomial regression equation that would take the same form as an adjusted Arrhenius equation describing the kinetics of the reaction.^[20]^ A response surface design is a setup for an experiment where for each regressor three points are taken, namely a lower boundary, the mid‐point and a higher boundary. Generally, for a face‐centered design, these boundaries are codified as −1, 0 and 1. In this work we were not interested in creating an abstract statistical model, but rather one with physical significance, therefore our factors remained un‐coded. With a polynomial fit of multiple regressors a “response surface” is created to facilitate finding an optimum. A response surface design is therefore usually performed after an initial factorial design with two‐level factors and linear fits. If the optimum is not found within the bounds of the factorial design it is followed by a “path of steepest ascent” approach. Instead of using this methodology to find an optimum, the main focus was put on mapping the effects of each regressor and constructing a physical equation from the dependence obtained.

A multiple polynomial regression analysis takes the form of a linear equation where each separate factor is included. In this sense, the *simplest first order* version with no quadratic terms and no interaction terms takes the following form:

(1) $$\;\hat{y}=\beta_{0}+\sum_{i}\beta_{i}{}^{*}x_{i}$$

Where ŷ is the actual (or expected) response, β_0_ is the coefficient corresponding to the intercept, β_i_ is the coefficient corresponding to the i‐th iteration of the regressor x_i_.

A physical meaning for a linear regression model can be established from the generalization of the formal reaction kinetics. Thus, in a general form for a given chemical process, the reaction rate *W* can be defined in a differential and logarithmic form:

(2) $$W=-\frac{d\left[X_{i}\right]}{v_{i}dt}=\frac{d\left[Y_{j}\right]}{v_{j}dt}=\prod_{i}kX_{i}^{n_{i}}$$

(3) $$ln\left(W\right)=\ln\left(k\right)+\sum_{i}n_{i}\ln X_{i}$$

The temperature dependence of the reaction rate constant is described by the Arrhenius equation:

(4) $$\ln\left(k\right)=-\frac{Ea}{R}{}^{*}\frac{1}{T}+ln\left(A_{k}\right)$$

Overall, the expansion of the rate constant in the rate equation leads to a full linear equation as such:

(5) $$\ln\left(W\right)=ln\left(A_{k}\right)-\frac{Ea}{R}{}^{*}\frac{1}{T}+\sum_{i}n_{i}\ln X_{i}$$

Via the comparative analysis of Equations (1) and (5), intercept β_0_ can be denoted as the frequency factor, and the rest are similar first order terms. Besides, one regressor (x_i_) may be seen as 1/T, leaving one coefficient β_i_ as −E_a_/R.

A second order polynomial regression of multiple variables includes square terms and interaction terms, each with its own coefficient.^[21]^ With the ordinary least squares method, empirical response values are fit to approximate the actual response. The fit is assessed by analyzing the *goodness of fit*. Additional values for assessing the model are the R^2^ value, adjusted R^2^ value, the predicted residual error sum of squares (PRESS), the predicted R^2^ and the p‐value assessing the statistical significance of each term in the model.^[22]^

A potential benefit of attempting to approximate a physical equation with an abstract statistical model is with the inclusion of normally excluded factors or terms, which might have a more complex effect on the reaction kinetics. This is exemplified by our addition of pressure as a factor, or the inclusion of *quadratic* and *interaction* terms showing the joint effect of couples of parameters. If the addition of these terms is properly justified, it can be indicative to some actual physical effects. The eventual *full regression equation* will take the form of

(6) $$\hat{y}=\beta_{0}+\sum_{i}\beta_{i}{}^{*}x_{i}+\sum_{i=n}\beta_{n}{}^{*}{x_{n}}^{2}+\sum_{i=m}\beta_{mjk}{}^{*}\left(x_{mj}{}^{*}x_{mk}\right)$$

Following this approach, the first step is to construct a full multiple polynomial regression with all quadratic terms and interaction terms. The resulting model is then examined and statistically insignificant terms or factors are removed using a stepwise elimination algorithm. To further justify the resulting model, the linearity of the corresponding main effects plot should be assessed along with the p‐value for the regressor.

The key process variables including temperature, hydrogen pressure, concentration of the catalyst and concentration of the base are modified into a logarithmic form to fit the physical equation (Table 1 *)*. Additionally, the corresponding responses are shown as the logarithm of the concentration of the reaction product divided by the reaction time which represents an averaged reaction rate *(*Table 1 *)*. It is worth noting that the average rate is different from the initial reaction rate conventionally used in kinetic experiments. The initial reaction rate is often preferred as it makes it easier to extract formal kinetic parameters. However, for fast processes and for processes with a more complex behavior extracting the initial rates can be problematic and the resulting values can be heavily impacted by the reaction initiation procedure. Besides, it does not provide information about the overall process kinetics which can change during the process. In contrast, the average reaction rate can be a good descriptor for obtaining the formal kinetic parameters and capturing more complex behavior. For the formal kinetic parameters, the assumption can be made that the rate throughout the reaction is either linear or in correlation with the initial reaction rate of the target process.

**Table 1:** Overview of the main variables used in all models, their mathematical notation and the form in which they are generally used

Variable	Notation	Input form
Temperature/T	X_1_	1/T
Pressure/P	X_2_	P
Catalyst concentration/(Cat)	X_3_	ln(Cat)
Base concentration/(Base)	X_4_	ln(Base)
Average reaction rate (system response)	y	ln(Product/Time)

The full quadratic fit was done in Matlab using linear regression model fit function (*fitlm*) based on a least squares methodology. The data is weighted with the *RobustOpts* function which uses an iteratively reweighted least squares methodology. The analysis of variance was done using the ANOVA procedure.^[23]^ This analysis was done on both the components of the model, and the whole model. R‐squared and adjusted R‐squared values are given with each produced model. The predicted R‐squared is produced by the following formula:

(7) $$pred\;R^{2}=1-\left(\frac{PRESS}{SST}\right)$$

where one is subtracted by PRESS (the prediction error sum of squares)^[4]^ which is calculated from all the factors in the model, divided by the SST (sum of square total).

**Figure 3:**
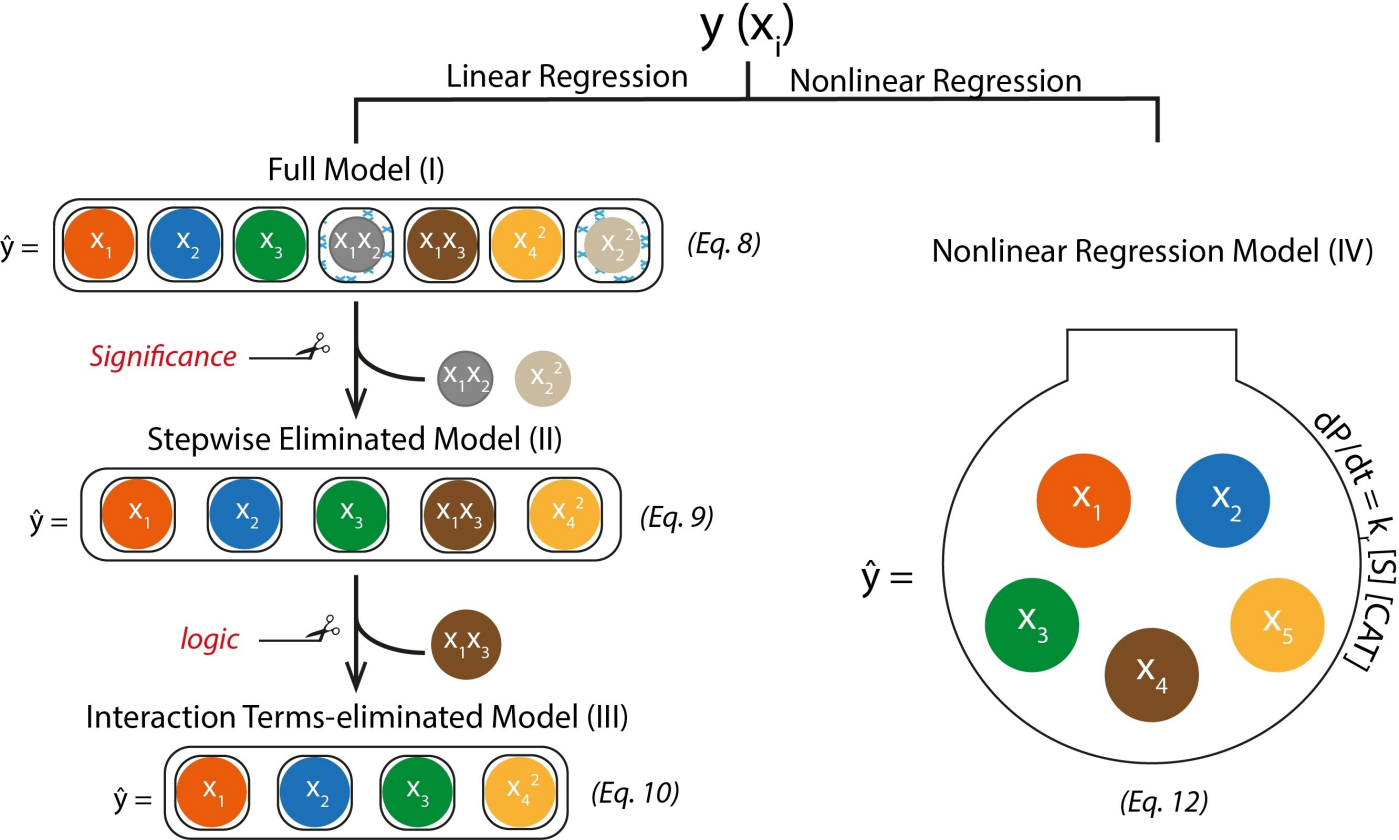
Statistical models based on linear (I–III) and non‐linear (IV) regression used in this work.

The data obtained from the catalytic tests were processed using four different statistic models schematically shown in Figure 3. First the full linear regression model (I) was constructed featuring all possible parameters and their interactions. Subsequent elimination of the parameters based on the statistical significance and chemical logic principles gave rise to the reduced linear regression models (II) and (III), respectively. The non‐linear regression model (IV) was constructed by following the general principles of the formal kinetics. The specific details of these models and the associated mathematical equations denoted in Figure 3 will be described in detail in the text below.

### Full Model (I)

To describe the experimental kinetic dataset, we first introduce kinetic model (I) based on the full multiple polynomial regression, which includes all the quadratic terms and interaction terms [Eq. (2)]. In this model the reaction rate (system response) can be defined as follows:

(8) $$\hat{y}=\;\beta_{0}+\beta_{1}x_{1}+\beta_{2}x_{2}+\beta_{3}x_{3}+\beta_{4}x_{4}+\beta_{5}x_{1}x_{2}+\beta_{6}x_{1}x_{3}+\beta_{7}x_{2}x_{3}+\beta_{8}x_{1}x_{4}+\beta_{9}x_{2}x_{4}+\beta_{10}x_{3}x_{4}+\beta_{11}{x_{1}}^{2}+\beta_{12}{x_{2}}^{2}+\beta_{13}{x_{3}}^{2}+\beta_{14}{x_{4}}^{2}$$

**Figure 4:**
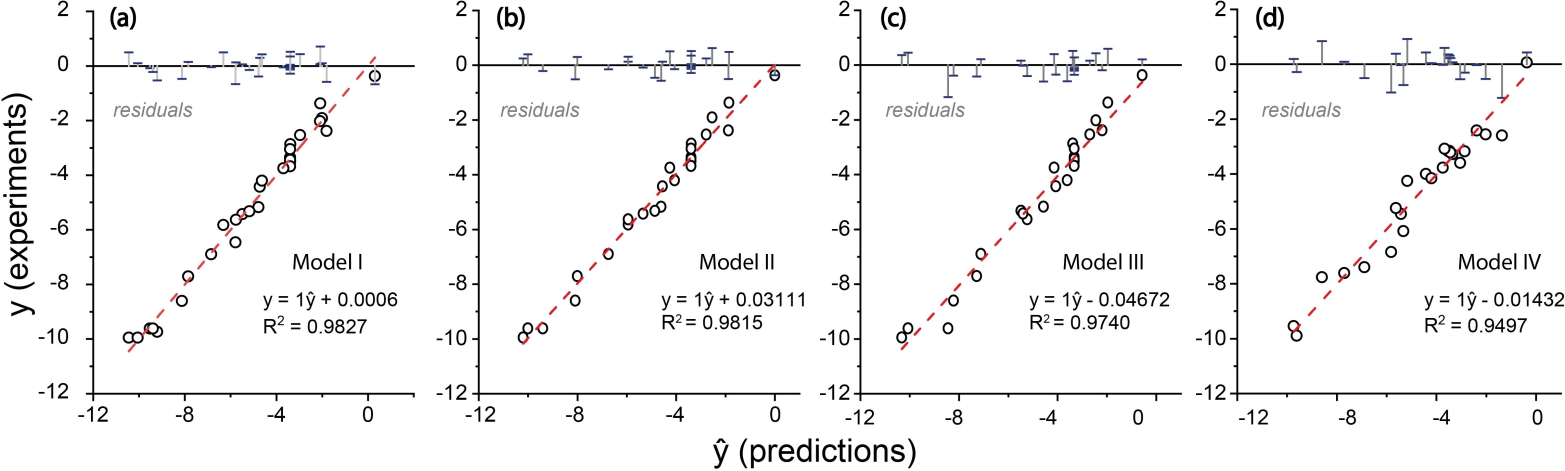
Model fitting with residual plot incorporated: a) full statistical model (I); b) stepwise eliminated model (II); c) interaction terms‐eliminated model (III); d) nonlinear regression model (IV). The y stands for the observed responses whereas the ŷ stands for the predicted response.

Figure 4 *a* shows the goodness of fit of the experimental data points with model (I). The results point to the substantial predictive power of this indiscriminately constructed multiple polynomial model as evidenced by pred‐R^2^=0.9003 (see *Table S3*). Further validation can be done via a p‐value test that gives the probability of new data deviating from model prediction (see *Table S4*). The p‐value of 1.29e^−10^ suggests a good predictability of this model based on the data provided. The F‐statistic vs. constant model was 60.7 showing the significance of the model against the model consisting of only a constant term. However, the analysis of the individual p‐values for each term in the model reveals many instances far larger than an α of 0.05 indicating that many of the term effects are to be deemed insignificant, or within the “noise” (see *Table S5*). The only terms deemed significant are x_1_, x_2_, x_3_, x_4_, the interactions x_1_ : x_3_ and x_2_ : x_4_, as well as the x_4_^2^ quadratic term. Furthermore, the p‐value for the lack of fit is 0.015 (see *Table S4*). These data show that the inclusion of excess (catalysis‐irrelevant) terms in model (I) renders it over‐fitted.

### Stepwise Eliminated Model (II)

To remove the redundant factors, a new model was derived using the stepwise regression methodology. This methodology iteratively adds terms and keeps them if they meet a pre‐defined criterion, or removes them otherwise. The criterion used was the p‐value for the F‐test of the change in the sum of squared error when a term is added or removed. Additionally, outliers were removed by assessing whether the standardized residuals were larger than 1.25 or smaller than −1.25. In our case, 4 outliers were determined, leaving a dataset of 26 observations. Accordingly, a model with significant terms only was obtained:

(9) $$\hat{y}=\;\beta_{0}+\beta_{1}x_{1}+\beta_{2}x_{2}+\beta_{3}x_{3}+\beta_{4}x_{4}+\beta_{5}x_{1}x_{3}+\beta_{6}x_{2}x_{4}+\beta_{7}{x_{4}}^{2}$$

This model has a predicted R^2^ of 0.9696 and an adjusted R^2^ of 0.978 (as can be seen i*n* Table 2). The nuance between predicted and adjusted R^2^ makes the assessing of these coefficients of determination trustworthy. The p‐value for the model being so small stems from this value being susceptible to overfitting. The goodness of fit is further exemplified by the residual plot (Figure 4 *b*).

**Table 2:** Statistical analyses for all the models comprising of statistical values including R‐squared, adjusted R‐squared, predicted R‐squared, the p‐value for the model, F‐statistic versus constant model, the root mean squared error (RMSE) and the p‐value for the lack of fit.

Models	R^2^	adj R^2^	pred R^2^	p‐value	F‐statistic vs. constant model	RMSE	Lack of fit p‐value
Model II	0.984	0.978	0.9696	6.75E‐15	160	0.387	0.0598
Model III	0.979	0.973	0.9572	2.24E‐14	165	0.413	0.0448
Model IV	0.955	0.945	0.9221	1.79E‐12	99.7	0.657	–

**Figure 5:**
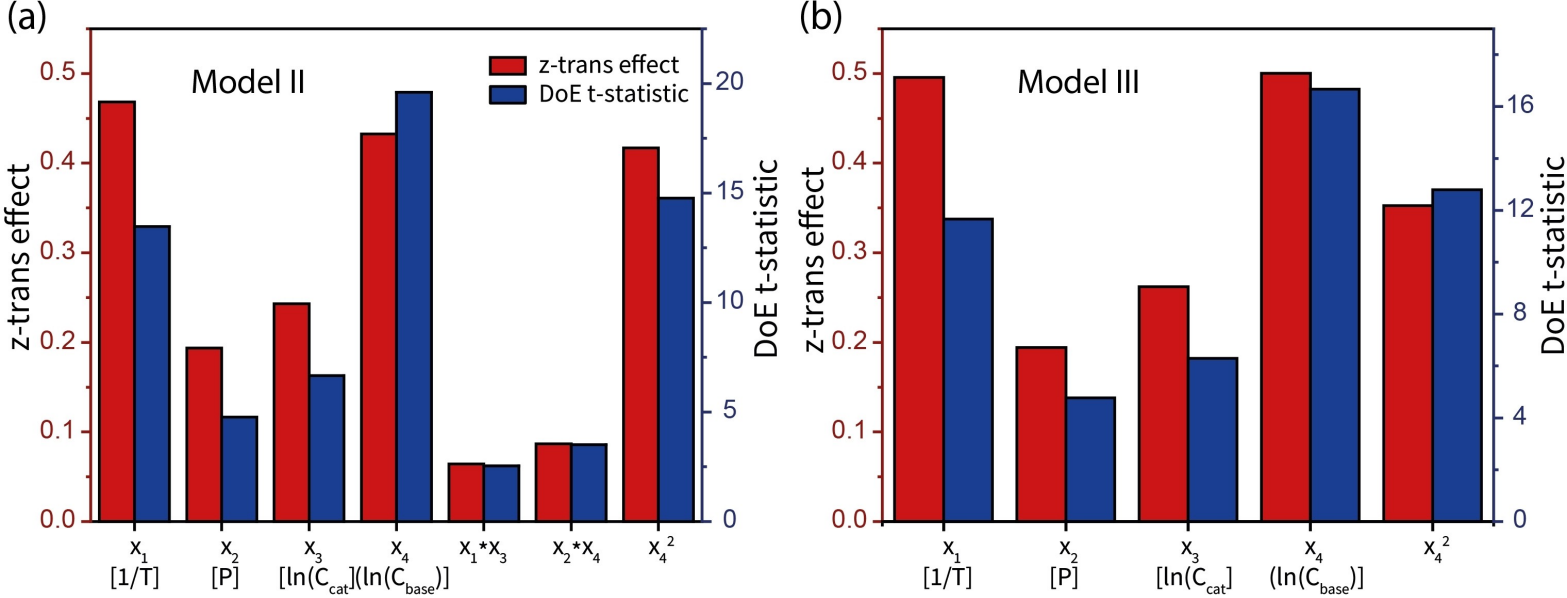
Effects of the reaction parameters: a) stepwise eliminated model (II); b) interaction terms‐eliminated model (III).

To further evaluate the models, the effect of model parameters was examined using z‐score and DoE t‐statistics methods. Upon conversion of each term value (including the response) to a “standard score” (also known as standardizing) or z‐score, with the same unit, we are able to compare the effects of those catalysis parameters.^[24]^ Besides, terms from multiple z‐transformed models can be compared.^[25]^ The z‐transformed effect results in Figure 5 *a* show that the terms with the largest weight are the temperature, the base concentration and the 2^nd^ order term for the base concentration.

The interaction terms have the lowest impact on the reaction rate. Next, by codifying the used factors as 1 and −1 for highest level and lowest level value respectively, the corresponding t‐statistic may be interpreted as the amount of influence of the term on the response in a standardized form, though the technical definition is it being a test of the null hypothesis that the effect is zero. The added value of this method is that the significance of such a standardized effect can be verified with a two tailed test using the error degrees freedom (df) and the chosen significance level α. For a df of 20 and an α of 0.05 the significance level is 2.086. In this case the response does not require standardization. The DoE t‐statistic results in Figure 5 *a* show that all the terms are significant, with the terms for the base concentration, the 2^nd^ order base concentration and the temperature having the most significance to the change in response being from these terms.

### Interaction Terms‐Eliminated Model (III)

The stepwise eliminated model also runs the risk of overfitting. Though the predicted R^2^ is excellent, the currently abstract statistical model must bear resemblance to reality. The interaction terms between temperature and the catalyst (x_1_x_3_, [Eq. (9)]), and the interaction term between the pressure and the base (x_2_x_4_, [Eq. (9)]) are not explained by the generalized rate equations. A new model was constructed by removing these interaction terms. The square term for the base however was kept due to the high weight of its effect. Whereas the first three primary parameters may be well fitted with a linear equation, the fourth parameter (the base) is undeniably second order. Interestingly, constructing the same stepwise model from this hypothetical rate normalized by the concentration of catalyst used (TOF) obviously eliminates the catalyst term and produces a model featuring only the main linear terms and a quadratic base term, and naturally excludes interaction terms. The regression equation for model III is similar but includes the catalyst term:

(10) $$\hat{y}=\;\beta_{0}+\beta_{1}x_{1}+\beta_{2}x_{2}+\beta_{3}x_{3}+\beta_{4}x_{4}+\beta_{5}{x_{4}}^{2}$$

All p‐values, except for that of the base term, are sufficiently low to refute the null hypothesis (see *Table S13*). Next, all experiments with standardized residuals higher than 1.25 and lower than −1.25 were removed from the model. This left 24 observations as 6 observations were identified as outliers. The residual plots for the resulting model (Figure 4c) show a good distribution but a bad normal probability fit (see *Figure S3*). The main effects of each parameter are presented as the prediction slice plots in the supporting information (see *Figure S4*). The prediction plot for the base concentration shows that at a high enough base concentration the corresponding effect saturates and reaches a plateau of a certain maximum rate.

Because the current model only leans on the validity of 24 remaining experiment data‐points, the p‐value for the lack of fit is only 0.0448 (Table 2). This shows that the terms currently used in the model are not sufficient. The missing terms could be the eliminated interaction terms, but could also be something as of yet unknown although the p‐value for the lack of fit can also be improved with more accurate additional data. Again, the results for the effects of the reaction parameters (Figure 5 *b*) show that the base concentration has the most significant effect on the reaction rate. Compared to the *z*‐score, however, we see that the quadratic term for the base concentration and the temperature have now exchanged places in the effect weight. With a *df* of 21 and an α of 0.05 the significance level is 2.080 (*t*‐test table).

### Nonlinear Regression Model (IV)

The linear regression models presented above imply that a kinetic equation, in which a linear product of the concentrations of the catalyst and the base produce the rate of alcohol production, which makes little sense from the chemical kinetics perspective. To understand why these variables give rise to such a good linear fit, the kinetics of the reaction were revisited. Though the exact reaction mechanism is not fully known, the basic process of catalyst activation and carbonyl compound hydrogenation can be explicitly considered. Following prior mechanistic analysis,^[19]^ we presume certainty over an elementary reaction where the catalyst is activated by the use of a base (e. g. potassium tert‐butoxide). Secondly, we assume certainty over the reaction, upon which the substrate is converted to the product with the use of the catalyst on a human time‐scale. No microscopic details are assumed for the catalytic process, but the respective processes are approximated by a power law instead. Therefore, the rate of the catalyst activation is

(11) $$\frac{d\left[CAT\right]}{dt}=k_{a}\left[{CAT}^{-}\right]\left[BASE\right]$$

where [CAT^−^] stands for the concentration of catalyst precursor and [CAT] is the concentration of the activated catalyst. Catalyst activation proceeds via the base‐assisted hydride formation step. Given the assumption of the semi‐batch catalytic experiment (*p*(H_2_)=constant during the reaction) with the solution saturated with H_2_, this component is left out of the reaction rate equation. The rate of the catalytic hydrogenation reaction can be written as

(12) $$\frac{dP}{dt}=k_{r}\left[S\right]\left[CAT\right]$$

where S and P denote the substrate (carbonyl derivative) and the product (alcohol) of the catalytic reaction. The reaction order for the catalyst and substrate will be introduced as coefficients in the statistic model. Integrating Equation 11 over time we obtain the following equation:

(13) $$\left[CAT\right]=\left[{CAT}^{0}\right]\left[{BASE}^{0}\right]e^{k_{a}t}$$

The zero in the superscript signifies the initial concentration. Combining Equations (12) and (13) gives:

(14) $$\frac{dP}{dt}=k_{r}\left[S\right]\left[{CAT}^{0}\right]\left[{BASE}^{0}\right]e^{k_{a}t}$$

Expanding k_r_ as dictated by the Arrhenius equation we obtain:

(15) $$\frac{dP}{dt}=Ae^{-Ea/RT}\left[S\right]\left[{CAT}^{0}\right]\left[{BASE}^{0}\right]e^{k_{a}t}$$

Taking the natural logarithm thereof gives:

(16) $$\ln\left(\frac{dP}{dt}\right)=\ln\left[A\right]-\frac{Ea}{R}{}^{*}\;\frac{1}{T}+ln\left[{CAT}^{0}\right]+ln\left[{BASE}^{0}\right]+ln\left[S\right]+k_{a}t$$

This equation resembles closely the one obtained from the stepwise elimination procedure [Eq. (9)], but with a few additional factors. The additional factors in this equation include ln[S] which is the natural logarithm of the substrate concentration, and *k_a_⋅t*, which is the catalyst activation rate constant times the time in seconds. The respective parameters were next estimated using a nonlinear regression model. Due to the data for rate being taken at the end of the reaction, a decision was made to use the substrate concentration at the end of the reaction (as measured by GC) and to use the reaction time in seconds that it took to finish each reaction according to the H_2_ consumption data.

The nonlinear model was set up as:

(17) $$\hat{y}=\beta_{0}-\left(\frac{\beta_{1}}{8.314{}^{*}10^{-3}}\right){}^{*}x_{1}+\beta_{2}x_{2}+\beta_{3}x_{3}+\beta_{4}x_{4}+x_{5}+\beta_{5}x_{6}$$

with x_2_ being pressure in bar, x_3_ being the natural logarithm of the catalyst concentration, x_4_ being the natural logarithm of base concentration, x_5_ being the natural logarithm of the substrate concentration at the end of the reaction (note there is no coefficient for this factor) and x_6_ being the reaction time in seconds and the corresponding coefficient β_5_ being the rate constant for catalyst activation.

This model is set up with six initial values of 1 for the coefficients, and with robust fitting options^[26]^ using weighted residuals tuned using the bi‐square weight function.^[27]^ Outliers were identified by setting the standardized residual at ±1.25 and removed. After removing the outliers, 24 observations are left and there are 19 error degrees of freedom. In table 2 certain statistical values for the model can be seen, like the R‐squared values for example. It must be said that R^2^ values cannot be used for nonlinear regression^[28]^ and should therefore be ignored.

Plotting the predicted rates with the experimental rates gives a sense for the goodness of fit with an R^2^ of 0.9497 (Figure 4d). It must be noted that the Jacobian of this model is ill‐conditioned, which can be attributed to the model being described by six independent factors.

**Figure 6:**
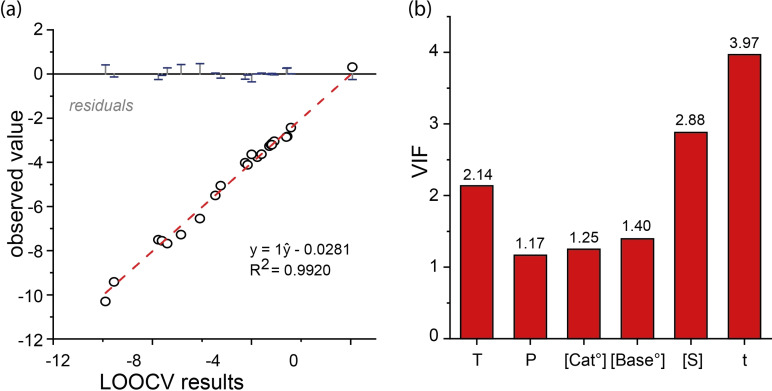
a) Leave‐One‐Out‐Cross‐Validation fit; b) Variance Inflation Factors (VIF) for all variables used in the nonlinear regression model.

A nonlinear regression model is generally more difficult to validate. One of the possible validation methods is “leave‐one‐out‐cross‐validation” (LOOCV). Upon analyzing the model using LOOCV, an RMSE of 0.732 is calculated and an R^2^ of 0.9920 (Figure 6 *a*).

All the p‐values for the coefficients in this model were found significant (see *Table S20*). The expansion of the model with an added coefficient for the substrate variable results in the respective p‐value of 0.24403 which is not significant. Importantly, the coefficients for most terms are comparable to the coefficients from the interaction eliminated model, saving for the obvious addition of the fifth coefficient related to the time term, and the base term which is now ∼1 potentially due to the lack of a second order term. The residual plots show a good distribution with a slight tailing in the histogram. The normal probability plot shows a good normal distribution and no residual sticks out in the residual vs fitted value plot or in the residual vs lagged residual plot (see *Figure S5*).

Assuming that this model accurately describes the reaction kinetics, it explains why the prior stepwise model fits well as it is in essence the stepwise model with additional factors corresponding to the substrate concentration and the reaction time. These factors are obviously related to the response as the substrate concentration is the current concentration at any given moment of time, and the reaction time is considerably dependent on how fast the reaction proceeds, which in itself depends on other parameters.

The Variance inflation factor (VIF) is a value that determines the multicollinearity of a term with other terms. Generally, a VIF of 4 would indicate strong multicollinearity, and require a term to be removed from the model. In the nonlinear model we see high VIF values for the substrate and the time terms, but low enough that one could argue they should remain (Figure 6 *b*). The high multicollinearity of these terms is due to the fact that the reaction time and substrate at the end of the reaction can be considered dependent variables.^[25]^

The main statistical characteristics of the different models constructed above are listed in Table 2. As it shows, the stepwise model (II) with the interaction terms shows a high predictability and could be considered pragmatic for predicting the results of future reactions within the model limits. Statistically, the population of observations is too low to make strong conclusions on the interaction terms as their effects are deemed small. The results in Table 2 show that the stepwise eliminated model has the lowest RMSE and the highest p‐value from the models considered here. The p‐value for the lack of fit is also the only one over 0.05 which indicates the model fits the data well. In contrast, the interaction terms eliminated model (III) has the best balance between overfitting, statistical accuracy and predictability. Its lack of fit p‐value is just barely insignificant with an alpha of 0.05. This however is likely due to the lack of degrees freedom, which can influence the calculation. As such, this model will be used to compare with the kinetic data obtained via conventional protocols. Interestingly, the equation [Eq. (17)] of nonlinear regression model (IV) shows high resemblance to those Equations (9) and (10) of linear stepwise multiple regression models (II, III). Accordingly, the statistical models (II, III) without incorporation of reaction mechanism should potentially be able to provide information on the kinetic nature of the reaction.

Stepwise eliminated model (II):

$$\hat{y}=\;\beta_{0}+\frac{\beta_{1}}{T}+\beta_{2}P+\beta_{3}ln\left[CAT\right]+\beta_{4}ln\left[BASE\right]+\frac{\beta_{5}}{T}\cdot ln\left[CAT\right]+\beta_{6}P\cdot ln\left[BASE\right]+\beta_{7}(ln{\left[BASE\right])}^{2}\;\left(18\right)$$

Interaction‐term eliminated model (III):

$$\hat{y}=\;\beta_{0}+\frac{\beta_{1}}{T}+\beta_{2}P+\beta_{3}ln\left[CAT\right]+\beta_{4}ln\left[BASE\right]+\beta_{5}(ln{\left[BASE\right])}^{2}\;\left(19\right)$$

Nonlinear regression model (IV):

$$\hat{y}=\beta_{0}-\left(\frac{\beta_{1}}{8.314{}^{*}10^{-3}}\right){}^{*}\frac{1}{T}+\beta_{2}P+\beta_{3}ln\left[CAT\right]+\beta_{4}ln\left[BASE\right]+ln\left[S\right]+\beta_{5}t\;\left(20\right)$$

Although the exact values of the main coefficients in the three considered model equations [Eq. (18), (19), (20)] vary significantly, pronounced similarities and common trends can be noted in the obtained parameters (Table 3).^[29]^ Most notably the models differ in the intercept values due to the regression equations being markedly different in the interdependence of each parameter. The coefficients for T^−1^ are similar for the interaction‐term eliminated model (III) and the nonlinear model (IV), but are distinctly different from that in the stepwise eliminated model (II). This is due to the fact that T^−1^ in the latter case is also accounted for the ln[CAT]⋅T^−1^ interaction term. Considering the coefficient for T^−1^ can represent activation energy Equations (16) and (17), the above result implies that the apparent activation energy is affected by initial catalyst concentration. Similarly, ln[CAT] has a decidedly higher coefficient in model II as compared to the other models (III and IV) because of the ln[CAT]⋅T^−1^ interaction term, suggesting effective catalyst concentration is limited by temperature. In both models II and III, the ln[BASE] is present with a negative coefficient. Model IV instead has a positive coefficient for ln[BASE], arguably due to the negative coefficient related to the reaction time t. This indicates a potentially negative coefficient for a parameter still missing in the equation IV. The coefficient for ln[BASE] thus can be interpreted as a potential catalyst deactivation path induced by base, as the reaction rate is seen to diminish at the higher [BASE] (see *Figure S4)*. It is also worth noting that interaction term [P]⋅ln[BASE] with a positive coefficient compensates for the negative effect of ln[BASE], suggesting high H_2_ pressure can suppress the aforementioned deactivation. The coefficients for the (ln[BASE])^2^ are equivalent between the linear models I and II. Finally, the coefficients for P are comparable in the three models.

**Table 3:** An overview of the parameter coefficients obtained from different statistical models.

Model parameters	Model II	Model III	Model IV
Intercept	57.28	16.705	31.554
T^−1[a]^	162.3	41.0	39.3
P	0.068951	0.040648	0.035241
ln[CAT]	6.0532	1.3553	1.5982
ln[BASE]	−2.6974	−2.1133	1.0932
ln[CAT]⋅T^−1^	−1601.3	–	–
P⋅ln[BASE]	0.0058	–	–
$(ln{\left[BASE\right])}^{2}$	−0.24092	−0.21516	–

[a] The coefficients for T^−1^ are normalized to the form that represent the apparent activation energy (kJ/mol).

### Model Validation & Kinetic Analysis

**Figure 7:**
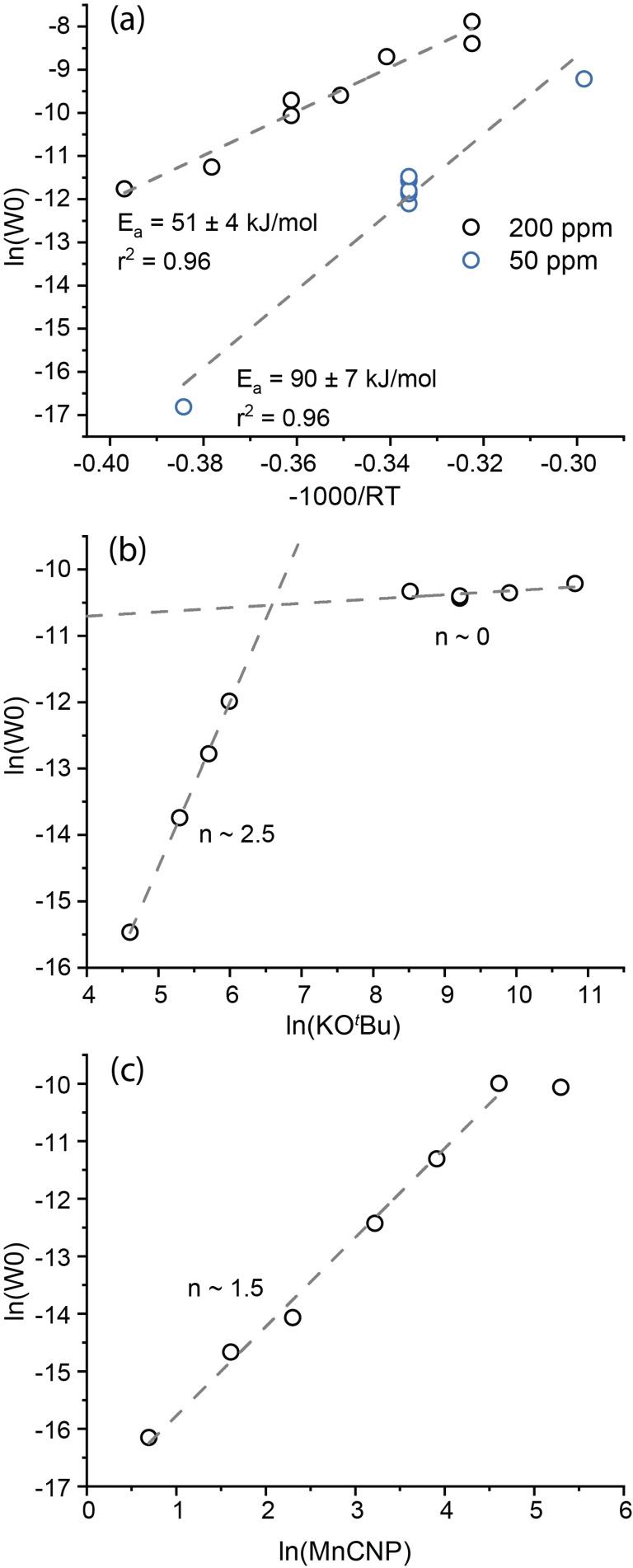
Kinetic data. a) Arrhenius plot for varying catalyst loadings showing the natural log of the rate constants vs the multiplicative inverse of the temperature multiplied by the gas constant. b) The rate order plot for bases concentration showing the natural log of the rate vs the natural log of base concentration. c) The rate order plot for catalyst concentration showing the natural log of the rate vs the natural log of the catalyst concentration.

The statistical analysis results were then compared to the reaction parameters obtained via conventional kinetic measurements. In formal kinetic experiments, the reaction parameters are extracted from the initial reaction rates (see Section 5 in the *SI*). The initial reaction rates for two different catalyst loadings were measured at varied temperature to assess the apparent activation energy, while kinetic runs at varying [BASE] and [CAT] were used to estimate the respective reaction orders. The results of the kinetic experiments are summarized in Figure 7.

The kinetic data shown in Figure 7 *a* reveal that already at the catalyst concentration of 200 ppm, the catalytic hydrogenation reaction is severely mass‐transfer limited. Indeed, for the higher catalyst concentration of 200 ppm we find an activation energy of 51 kJ/mol that is approximately 2 times lower than the value obtained at a lower (50 ppm) catalyst concentration. The change in the catalyst reaction order at higher loading also supports this (Figure 7 *c)*. Our data strongly imply that, with mass‐transfer limitations, the intrinsic activity of catalysts can be missed when screening them even at a relatively low loading amount (e. g. 200 ppm). In the intrinsic kinetics regime, the reaction order in the catalyst was close to 1.5, while the reaction order for the base changes from ca. 2.5 at low base concentration to 0 at high concentrations (Figure 7b). The complex behavior observed for these kinetic parameters suggest that the reaction has different regimes.

An increased reaction rate at high catalyst loading might result in diffusion limitations, while the initial reaction rate can be affected by the catalyst activation processes. It is worth noting that the exact role of the base in the homogeneous hydrogenation processes is not fully clear and might play an important role in both pre‐catalyst activation and deactivation processes.^[19]^ Furthermore, previous studies have shown that the KO^*t*^Bu cation may facilitate the ketone hydrogenation by functioning as a Lewis acid.^[7e]^ In our previous work we have shown direct hemilability of the phosphine arm when KBHEt_3_ was used as an alternative activator. Similarly, the hemilability can play an important role when KO^*t*^Bu is used as the base.^[19]^

Next, the reaction parameters obtained from the direct kinetic measurements were compared to the results of the statistical analysis. The comparison of model III and IV with the barriers obtained from the kinetic measurements (Figure 7 *a*) reveal a close coherence of the T^−1^ coefficient of ∼41 kJ/mol and ∼39 kJ/mol with the Arrhenius barrier (Table 3). Furthermore, the ln[CAT] coefficients derived from both models III and IV produced similar catalyst orders of ∼1.35 and ∼1.59 (Table 3). Similarly, we observed the same comparableness with the base concentration, as the coefficient corresponding to the ln[BASE] in model III is −2.11. The absolute value would be comparable to the ∼2.5 rate order found in the rate order plot for the base. However, it must be noted that the parameters have different signs. This is likely due to the completely different effects captured by the statistical and kinetic analysis.

While conventional kinetics measured under the differential conditions gives information about the initial stages of the reaction, statistical analysis is focused on the effect of the reaction parameters on the average reaction rate. Thus, the increased base concentration can have a strong positive effect on the pre‐catalyst activation resulting in a higher initial reaction rate. On the other hand, it can contribute to the catalyst deactivation at the later stages of the reaction giving rise to a decreased final yield and, accordingly, lower average rate. The nonlinear regression model IV also shows resemblance between statistical coefficients and direct kinetic measurements. The activation rate from this model is ∼39 kJ/mol which corresponds better to the ∼41 kJ/mol value from model III than to the 51 kJ/mol from the experimental results. The coefficient corresponding to the catalyst concentration is 1.6 which neatly corresponds to the ∼1.5 rate order from kinetic experiment. Other parameters however diverged. Thus, the coefficient corresponding to the base concentration is ∼1.1 which is markedly different from the ∼2.5. When the stepwise model is made without quadratic term a similar coefficient (∼0.9) was found. The coefficient of ∼1 could be due to the lack of a quadratic term, whereas the fit remains acceptable due to the (over)abundance of terms in the form of time and substrate concentrations. This potential overabundance is indicated by an ill‐conditioned Bayesian.

## Conclusion

The kinetics of homogeneous hydrogenation of benzophenone ketone substrate catalyzed by a recently developed highly active Mn(I)‐CNP catalyst has been investigated and analyzed in the framework of formal kinetics as well as by using a response surface Box‐Wilson statistical methodology. In addition to gaining deeper insights into the behavior of this highly potent catalytic system, the study aimed at investigating the possibility of enhancing the data output from high‐throughput catalyst screening/optimization procedures. The results presented highlight the critical role of the secondary effects such as the reaction temperature and the presence and concentration of base activator/promotor on the performance of the homogeneous carbonyl hydrogenation catalysts.

Equating the regression equations and the coefficients derived from it with statistical values has to be treated with strong skepticism and must therefore require rigorous statistics to validate this kind of method. The exact relation of the statistical models to the intrinsic reaction kinetics is often difficult to evaluate. However, having found significant models, we are now able to speculate on the relations of certain factors to the observed reaction rate. In this work the stepwise eliminated model (II) is found to be the most statistically significant. The interaction terms between temperature and catalyst concentration and between the pressure and the base concentration indicate some complexity worth further investigation. The interaction terms eliminated model (III) retains a quadratic term for the base concentration, which may be related to the complex role that the base plays in the reaction. For both of the models II and III we consistently find that the base concentration holds the strongest effect on the reaction rate. The limited effect we observe from the pressure is explainable by the increase in molecular hydrogen availability within the reaction medium. We have clearly demonstrated the ability of the statistical model to measure the activation energy of the reaction and to capture different reaction regimes. The coefficients resulting from the stepwise model correlate with the kinetic parameters from classically obtained kinetics. Additionally, the nonlinear model (IV) can be compared with the addendum that an argument could be made for the removal of substrate and time due to the fairly high VIF value, which in turn explains why models II and III are valid.

The statistical methodology described in this paper may prove a reliable method to further understand complex and seemingly chaotic reactions. An apparent advantage of such an approach is that no initial assumption for the kinetics or the reaction mechanisms are required. This provides an opportunity to construct descriptive yet unbiased models. Besides it allows for the simultaneous testing of multiple factors allowing understanding of how one factor may influence the other, the addition of terms that usually do not belong to a “default” kinetic rate equation in order to understand the effect this term has, the statistical validation of the data and any coefficients or conclusions taken thereof and allowing extensive interpolation and predictability (effectively mapping a response to multiple factors of interest). Additionally, once the model is made it can be used for both optimization and for mechanistic studies. A careful marriage between reaction kinetics and statistical modelling could prove useful for combining the chemical fundamentals of the reaction mechanism happening in reality with our theoretical understanding of the reaction mechanism, usually supported by computational chemistry and through the avenue of microkinetic modelling.

## Experimental Section

Acetophenone hydrogenation was chosen as a model reaction. The reaction schematics, catalyst structure and the experimental setup are shown in Figure 2. Catalytic runs were performed using a home‐built parallel high‐pressure low‐volume autoclave system equipped with pressure sensors. In a typical catalytic run, the reaction mixture containing substrate (acetophenone, 5 mmol), potassium tert‐butoxide (KO^*t*^Bu), catalyst (MnCNP), solvent (dioxane) and an internal standard (dodecane) were charged in the autoclave inside a moisture‐ and air‐free glove box. Prior to experiments, all liquid chemicals were treated with basic alumina, degassed in a Schlenk line and dried over 3 Å molecular sieves. The autoclaves were then sealed inside the glovebox and connected to the gas feeding system of the catalytic setup. All gas lines were purged with compressed Ar to remove air, and then the reactors were pressurized to a target pressure with hydrogen gas. The reaction mixtures were stirred with Teflon‐coated magnetic stirrers and rapidly heated to a target reaction temperature. Prior to experiments, the temperature in the reactors was calibrated using an external thermocouple. Molar hydrogen consumption during the course of the reaction was followed with parallel pressure sensors volumetrically calibrated prior to catalytic experiments. After the reaction, products were analyzed with a gas chromatograph equipped with an FID detector.

## Conflict of interest

The authors declare no conflict of interest.

## Supporting information

As a service to our authors and readers, this journal provides supporting information supplied by the authors. Such materials are peer reviewed and may be re‐organized for online delivery, but are not copy‐edited or typeset. Technical support issues arising from supporting information (other than missing files) should be addressed to the authors.

Supporting Information
