# Supplementary material for: Utilizing Design of Experiments Approach to Assess Kinetic Parameters for a Mn Homogeneous Hydrogenation Catalyst
Source: ChemCatChem. 2021 Sep 14;13(23):4886–96. doi: 10.1002/cctc.202101140 (PMC9291086; doi:10.1002/cctc.202101140)
Supplement: Supplementary file 1 — Supporting Information [file CCTC-13-4886-s001.pdf]

ChemCatChem

Supporting Information

Utilizing Design of Experiments Approach to Assess Kinetic Parameters for a Mn Homogeneous Hydrogenation Catalyst

Robin K. A. van Schendel[+], Wenjun Yang[+], Evgeny A. Uslamin,* and Evgeny A. Pidko*

1. Catalyst characterization[1]

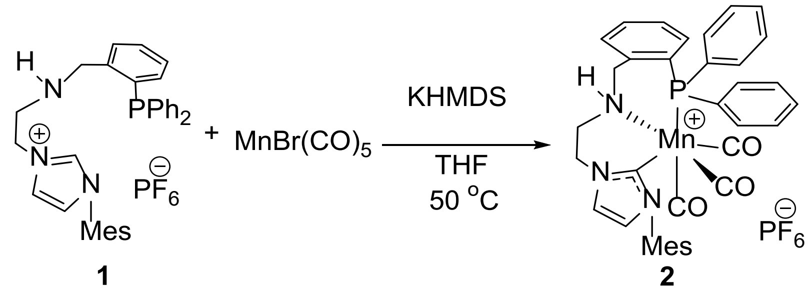

To the orange suspension of $[MnBr(CO)_5]$ (137.5 mg, 0.5 mmol) in THF (5 mL) was added ligand **1** (325 mg, 0.5 mmol) and stirred for 3 h at 50 ºC. The resulting solution was then cooled down to room temperature, potassium bis(trimethylsilyl)amide (110 mg, 0.55 mmol) in THF (2 mL) was added dropwise, stirred for another 3 h and evaporated to dryness. Residual oil was washed with pentane, re-dissolved in DCM, filtered through a Celite plug to remove inorganic salts and evaporated to dryness. The crude product was further purified by crystallization (diethyl ether vapor diffusion into solution in THF) to afford **2** as yellow solid in 51% yield (200.1 mg).

2. General procedure of Catalytic Hydrogenation:

Liquid substrates were passed through a plug of neutral alumina, degassed and stored over molecular sieves in the glove box. Mn catalysts, KBHEt3 and KOtBu, substrates and solvents were handled in the glovebox. Kinetic study were done with a autosampler setup.3For initial experiments and optimisation, products were analyzed on an Agilent 6890 gas chromatograph equipped with an FID detector. Method details: Agilent CP-Chirasil-Dex CB column (25 m, 0.25 mm ID, 0.25 μm film thickness) and temperature profile from 120 °C (hold 1 min) up to 180 °C with ramp of 20 °C/min and hold at 180 °C for 2 minutes. Mass balances were verified to be within 90 % – 110 % for all experiments. Products were identified using retention times and peak areas from analytically pure reference samples. The real-time hydrogen pressure during

hydrogenations was recorded and transformed to corresponding H_2 consumption (mmol) via ideal gas law (PV = nRT).

2.1. Catalytic hydrogenation with Mn catalyst:

Stock solutions of the catalyst (0.01 M) were prepared. KOtBu (5.6 mg, 1 mol%), substrate (5 mmol), solvent (3 mL), dodecane (56.8 µL, 0.25 mmol), Mn complex (250, 500, 1000 µL for S/Mn 20000, 10000, 5000, 4000 respectively) were mixed into a 4 mL glass vials and transferred into a stainless steel autoclave in the glovebox. The system was purged with N_2 (3×8 bar) and H_2 (1×30 bar), pressurized with H_2 to specified pressure, and heated to specified temperature. After the reaction, resulting mixture was quenched with HCl aqueous (50 µL, 1 M) and then GC samples were prepared by dilution of the reaction mixture in THF (20 µL into 1 mL THF).

3. Design of experiment

Table S1. The parameters used in experiments and their corresponding reaction time, overall reaction rate and yield

T, °C	Concentration, mmol/l		Pressure (bar)	Time (hours)	Alcohol mol/h	Yield (%)
	Catalyst	Base (KOtBu)				
65	0.16	6.28	30	15.51	0.030944052	37.22
65	0.16	6.28	30	15.51	0.029359893	36.20
65	0.16	6.28	30	15.51	0.034994558	43.00
65	0.16	6.28	30	15.51	0.032121668	39.45
65	0.16	6.28	30	15.51	0.025267823	30.90
65	0.06	6.28	30	15.51	0.005671168	6.98
65	0.16	6.28	50	7.97	0.148097992	94.83
65	0.16	12.50	30	15.44	0.056845424	70.24
65	0.16	6.28	10	15.44	0.023622165	29.17
65	0.25	6.28	30	14.47	0.079785331	92.69
65	0.16	6.28	30	15.44	0.047355875	58.48
65	0.16	0.06	30	15.44	0.000183823	0.23
100	0.25	12.50	50	1.67	0.694090492	92.34
100	0.06	12.50	50	8.31	0.132726873	87.64
100	0.25	12.50	10	12.22	0.092152914	89.34
100	0.25	0.06	10	18.47	0.002970152	4.36
100	0.06	0.06	50	18.47	0.000451125	0.66
100	0.25	0.06	50	18.47	0.004409259	6.44
100	0.06	12.50	10	22.43	0.01199248	21.40
100	0.06	0.06	10	22.43	5.90626E-05	0.11
100	0.16	6.28	30	4.64	0.253809451	92.84
30	0.06	0.06	10	27.78	4.76931E-05	0.11
30	0.25	12.50	10	27.78	0.003580893	7.93
30	0.06	12.50	50	27.78	0.004869708	10.78
30	0.06	0.06	50	27.78	4.76931E-05	0.11
30	0.25	12.50	50	27.78	0.014979701	33.18
30	0.06	12.50	10	27.78	0.001012782	2.24
30	0.25	0.06	50	19.80	6.69095E-05	0.11
30	0.25	0.06	10	19.80	6.69095E-05	0.11
30	0.16	6.28	30	19.80	0.001570081	2.48

Table S2. The parameters used in the models, transformed from the values in Table S1, and the corresponding transformed reaction rate.

1/T	Pressure, bar	ln(mol/l catalyst)	ln(mol/l base)	ln(mol/l alcohol per h)
0.002949	30	-8.76	-5.07	-3.48
0.002949	30	-8.76	-5.07	-3.53
0.002949	30	-8.76	-5.07	-3.35
0.002949	30	-8.76	-5.07	-3.44
0.002949	30	-8.76	-5.07	-3.68
0.002949	30	-9.68	-5.07	-5.17
0.002949	50	-8.76	-5.07	-1.91
0.002949	30	-8.76	-4.38	-2.87
0.002949	10	-8.76	-5.07	-3.75
0.002949	30	-8.29	-5.07	-2.53
0.002949	30	-8.76	-5.07	-3.05
0.002949	30	-8.76	-9.68	-8.60
0.002673	50	-8.29	-4.38	-0.37
0.002673	50	-9.68	-4.38	-2.02
0.002673	10	-8.29	-4.38	-2.38
0.002673	10	-8.29	-9.68	-5.82
0.002673	50	-9.68	-9.68	-7.70
0.002673	50	-8.29	-9.68	-5.42
0.002673	10	-9.68	-4.38	-4.42
0.002673	10	-9.68	-9.68	-9.74
0.002673	30	-8.76	-5.07	-1.37
0.003288	10	-9.68	-9.68	-9.95
0.003288	10	-8.29	-4.38	-5.63
0.003288	50	-9.68	-4.38	-5.32
0.003288	50	-9.68	-9.68	-9.95
0.003288	50	-8.29	-4.38	-4.20
0.003288	10	-9.68	-4.38	-6.90
0.003288	50	-8.29	-9.68	-9.61
0.003288	10	-8.29	-9.68	-9.61
0.003288	30	-8.76	-5.07	-6.46

4. Statistics

All statistical models were made using Matlab R2016a Version: 9.0.0.341360. Goodness of fit plots were made in Excel.

4.1. Full statistical model (I)

The full statistical model was made using the variables and response in table S2. The variables were titled “Data”, and the response was titled “Response”. Using the function “fitlm(Data, Response, 'quadratic')”, a full polynomial regression model was made. Using the “anova” function with both “component” and “summary” arguments provides analyses of variance for each term or for the entire model. The PRESS value was calculated using the script from Trujillo-Ortiz,[2] but required a data list of all the term values that take part in the regression equation. This list had to be manually made. The predicted R-squares were calculated by dividing the PRESS by the SST of the model and subtracting this from a value of 1. Residual plots were made using the “plotResiduals” function. The default plot is a histogram of residuals. Additional plots like the normal probability plot, the residuals vs fitted values plot and the residuals vs lagged residuals plot are obtained by using the arguments ‘probability’, ‘fitted’ and ‘lagged’ respectively.

Table S3. Estimated Coefficients for full polynomial regression model and corresponding statistical values like the square error, t-statistic and the p-value.

	Estimate	SE	tStat	pValue
(Intercept)	12.92	60.033	0.21522	0.83249
x_1	-2933.4	21213	-0.13828	0.89186
x_2	0.022821	0.11496	0.19851	0.84531
x_3	0.4413	13.664	0.032296	0.97466
x_4	-1.122	1.7074	-0.65712	0.52106
x_1:x_2	-39.281	20.924	-1.8773	0.080063
x_1:x_3	-2156.1	600.48	-3.5906	0.0026766
x_1:x_4	-271.31	153.41	-1.7685	0.097296
x_2:x_3	-0.00953	0.0092376	-1.0319	0.31847
x_2:x_4	0.006024	0.0023601	2.5522	0.022102
x_3:x_4	0.004032	0.06768	0.059569	0.95329
x_1^2	-3.72E+06	3.42E+06	-1.0872	0.29413
x_2^2	0.00137	0.00080019	1.712	0.10749
x_3^2	-0.41955	0.74968	-0.55964	0.58399
x_4^2	-0.19002	0.10492	-1.811	0.090208

Number of observations: 30, Error degrees of freedom: 15

Root Mean Squared Error: 0.515

R-squared: 0.983, Adjusted R-Squared 0.966

F-statistic vs. constant model: 60.7, p-value = 1.29e-10

PRESS: 22.8951, Predicted R-Squared 0.9003

Table S4. Analysis of Variance (ANOVA) of the full polynomial regression model.

	SumSq	DF	MeanSq	F	pValue
Total	229.53	29	7.915		
Model	225.56	14	16.111	60.743	1.29E-10
. Linear	215.21	4	53.801	202.85	7.32E-13
. Nonlinear	10.35	10	1.035	3.9022	0.0089328
Residual	3.9785	15	0.26523		
. Lack of fit	3.7553	10	0.37553	8.4128	0.014863
. Pure error	0.22319	5	0.044638		

Table S5. Analysis of Variance (ANOVA) of the terms of the full polynomial regression model.

	SumSq	DF	MeanSq	F	pValue
x_1	45.147	1	45.147	170.22	1.37E-09
x_2	7.5901	1	7.5901	28.617	8.11E-05
x_3	13.997	1	13.997	52.772	2.77E-06
x_4	112.84	1	112.84	425.45	2.02E-12
x_1:x_2	0.93476	1	0.93476	3.5243	0.080063
x_1:x_3	3.4196	1	3.4196	12.893	0.0026766
x_1:x_4	0.82958	1	0.82958	3.1277	0.097296
x_2:x_3	0.28243	1	0.28243	1.0648	0.31847
x_2:x_4	1.7277	1	1.7277	6.5137	0.022102
x_3:x_4	0.00094118	1	9.41E-04	0.0035485	0.95329
x_1^2	0.31348	1	0.31348	1.1819	0.29413
x_2^2	0.77736	1	0.77736	2.9309	0.10749
x_3^2	0.083069	1	0.083069	0.31319	0.58399
x_4^2	0.86992	1	0.86992	3.2798	0.090208
Error	3.9785	15	0.26523		

Yhat or $\hat{y}$ is the theoretical predicted response value calculated using the regression equation. These are then compared in the goodness of fit plot to y, or the empirical experimental values obtained in the lab. These are the natural logarithm of the hypothetical reaction rate.

Table S6. Values corresponding to the goodness of fit (Figure 3a in manuscript) plot of the full polynomial regression model.

yhat	y
-3.39858	-3.47557
-3.39858	-3.52813
-3.39858	-3.35256
-3.39858	-3.43822
-3.39858	-3.67822
-4.78667	-5.17236
-2.00711	-1.90988
-3.38524	-2.86742
-3.69413	-3.74557
-2.95994	-2.52842
-3.39858	-3.05006
-8.12942	-8.60154
0.303333	-0.36515
-2.08792	-2.01946
-1.80394	-2.38431
-6.31661	-5.81914
-7.84758	-7.70377
-5.48594	-5.42405
-4.72377	-4.42348
-9.20683	-9.73691
-2.08278	-1.37117
-10.4475	-9.95072
-5.76685	-5.63214
-5.17891	-5.32472
-10.0545	-9.95072
-4.62588	-4.20106
-6.84845	-6.89505
-9.5311	-9.61217
-9.39546	-9.61217
-5.79079	-6.45663

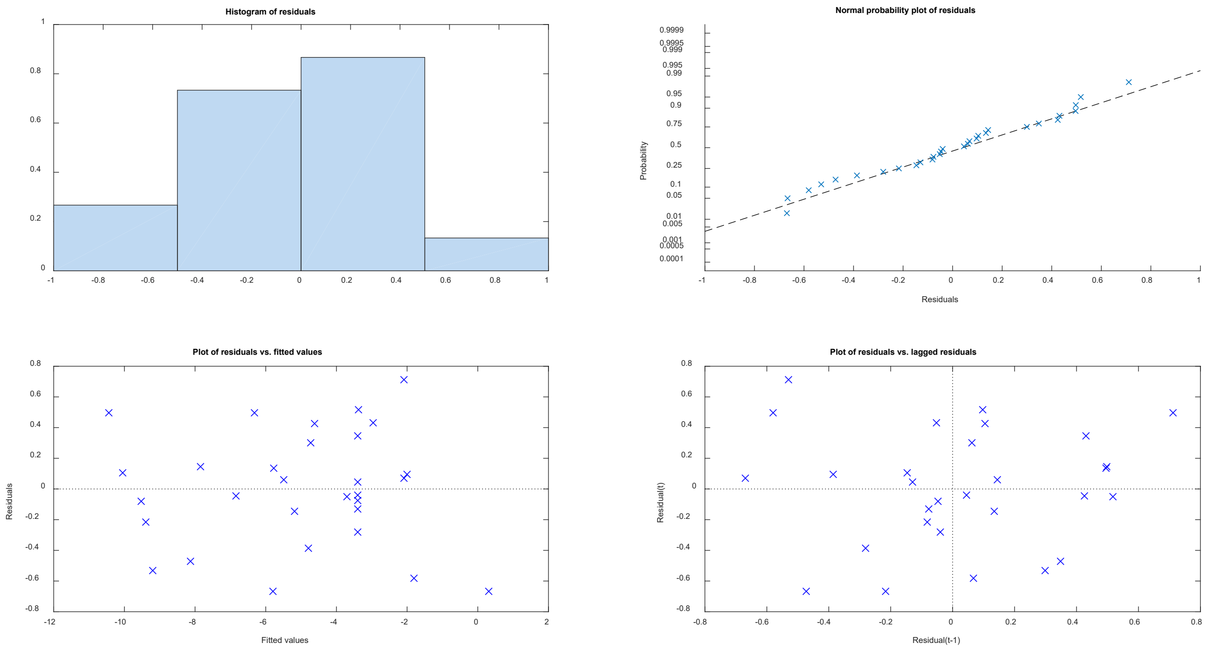

Figure S1. Assortment of residual plots corresponding to the full polynomial regression model. Top left: Histogram of residuals. Top right: Normal probability plot of residuals. Bottom left: residual plot. Bottom right: Lagged residual plot.

4.2. Stepwise eliminated model (II)

The stepwise eliminated model was made using the same dataset as the full model. The model was made using the "stepwiselm" function using the argument "SSE" as criterion. This criterion evaluates the p value for an F-test of the change in the SSE as the term is removed or added. The "quadratic" argument was used to make sure the model had the potential of being a full polynomial regression model if all terms had been significant. Using this model, first all p-values for the terms were assessed to be above 0.5, after which outliers were eliminated by taking out all data points with a standardised residual above 1.25, and then all data points with a standardised residual below -1.25. This method identified run 14, 20, 22 and 30 as outliers. A new model was made using the same function and the same arguments with an added input argument "exclude". The data list for calculating the PRESS only included the variables, the interaction terms used in the regression equation of this model, the quadratic term for the base and the response. Using a for-loop, a new data list without the outliers was made of the data list so that the PRESS value would be calculated using the data set actually used in the model (so without the outliers). The residual plots were made the same way as with the full model.

Table S7. Estimated Coefficients for stepwise eliminated regression model and corresponding statistical values like the square error, t-statistic and the p-value.

	Estimate	SE	tStat	pValue
(Intercept)	57.28	14.22	4.0283	0.00078861
x1	-19523	4674	-4.177	0.00056646
x2	0.068951	0.014934	4.6171	0.00021405
x3	6.0532	1.6052	3.771	0.001399
x4	-2.6974	7.76E-01	-3.4744	0.002706
x1:x3	-1601.3	529.76	-3.0226	0.0073158
x2:x4	0.0057967	0.00217	2.6712	0.015574
x4^2	-0.24092	0.054258	-4.4402	0.00031611

Number of observations: 26, Error degrees of freedom: 18

Root Mean Squared Error: 0.387

R-squared: 0.984, Adjusted R-Squared 0.978

F-statistic vs. constant model: 160, p-value = 6.75e-15

PRESS: 5.1862, Predicted R-Squared: 0.9696

Table S8. Analysis of Variance (ANOVA) of the stepwise eliminated regression model.

	SumSq	DF	MeanSq	F	pValue
Total	170.39	25	6.8157		
Model	167.7	7	23.957	160.08	6.75E-15
. Linear	161.96	4	40.49	270.56	8.27E-16
. Nonlinear	5.7378	3	1.9126	12.78	0.00010313
Residual	2.6938	18	0.14966		
. Lack of fit	2.4706	13	0.19005	4.2575	0.0598
. Pure error	0.22319	5	0.044638		

Table S9. Analysis of Variance (ANOVA) of the terms of the stepwise eliminated regression model.

	SumSq	DF	MeanSq	F	pValue
x1	37.282	1	37.282	249.12	5.48E-12
x2	5.5442	1	5.5442	3.70E+01	9.44E-06
x3	9.1836	1	9.18E+00	61.365	3.30E-07
x4	108.18	1	108.18	7.23E+02	5.53E-16
x1:x3	1.3673	1	1.3673	9.14E+00	0.007316
x2:x4	1.0679	1	1.0679	7.14E+00	0.015574
x4^2	2.9505	1	2.9505	19.715	0.000316
Error	2.6938	18	0.14966		

Table S10. Values corresponding to the goodness of fit plot (Figure 3b in manuscript) of the stepwise eliminated regression model.

Run	yhat	y
1	-3.39834	-3.47557
2	-3.39834	-3.52813
3	-3.39834	-3.35256
4	-3.39834	-3.43822
5	-3.39834	-3.67822
6	-4.60785	-5.17236
7	-2.53989	-1.90988
8	-3.38723	-2.86742
9	-4.25679	-3.74557
10	-2.77793	-2.52842
11	-3.39834	-3.05006
12	-8.08687	-8.60154
13	-0.00225	-0.36515
15	-1.88425	-2.38431
16	-5.94836	-5.81914
17	-8.00621	-7.70377
18	-5.33743	-5.42405
19	-4.55302	-4.42348
21	-1.85917	-1.37117
23	-5.94763	-5.63214
24	-4.86524	-5.32472
25	-10.2004	-9.95072
26	-4.06564	-4.20106
27	-6.74723	-6.89505
28	-9.40081	-9.61217
29	-10.0117	-9.61217

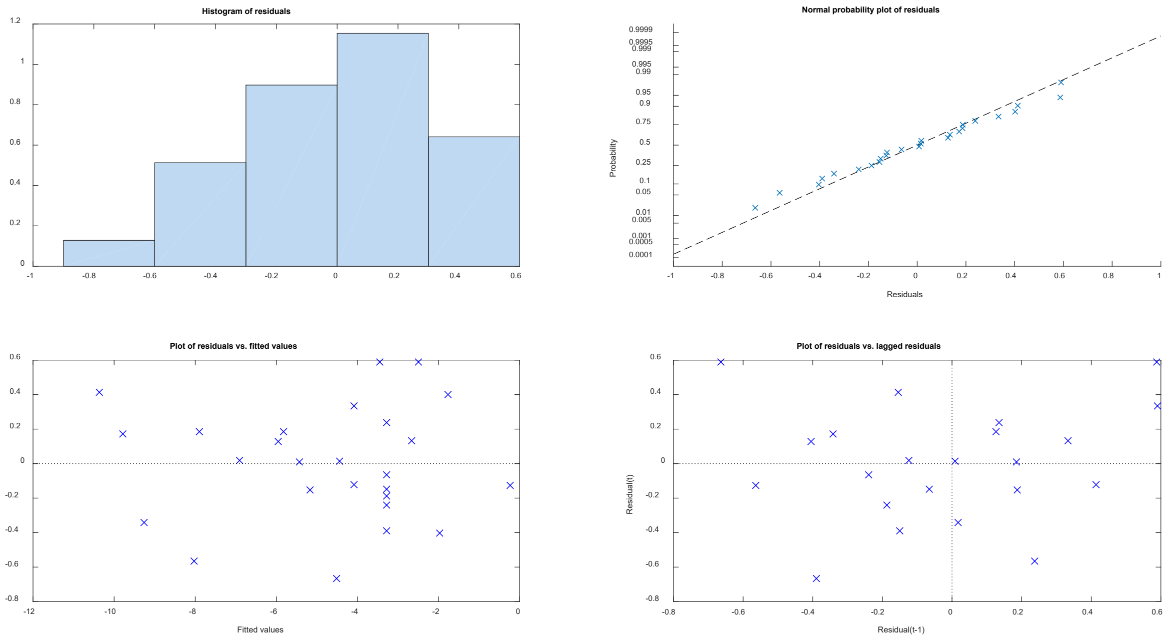

Figure S2. Assortment of residual plots corresponding to the stepwise eliminated regression model. Top left: Histogram of residuals. Top right: Normal probability plot of residuals. Bottom left: residual plot. Bottom right: Lagged residual plot.

Stepwise eliminated model main effects

The z-transformation effect sizes were obtained by subtracting variable value by the mean of that variable and dividing this by standard deviation of that variable. The same was done with the response. The resulting standardised variables were then used to make the model. The absolute estimated coefficients were the main effect sizes.

Table S11. Main effect sizes as estimated coefficients from a stepwise eliminated regression model built from z-transformed variables and response.

	effects
temperature * catalyst	0.064382
pressure * base	0.086802
pressure	0.19377
catalyst	0.24304
base ^2	0.41686
base	0.43244
temperature	0.46833

The DoE main effect sizes were obtained by using the regular variables, meaning not the inverse of the temperature and not the natural log of the concentrations. These variable values were then normalised using the following formula (where X_{DoE} is the normalised variable, X is the variable value, X_{min} is the lowest variable value and X_{max} is the highest variable value).

$$X_{DoE} = \frac{2 * (X - X_{min})}{X_{max} - X_{min}} - 1$$

With the normalised values but the original response, the model was made and the absolute T-statistic were the main effect sizes.

Table S12. Main effect sizes as T-statistic from a stepwise eliminated regression model built from DoE normalised variables.

	T-statistic
temperature * catalyst	2.5415
pressure * base	3.5054
pressure	4.7654
catalyst	6.6608
temperature	13.464
base ^2	14.766
base	19.603

4.3.Interaction terms eliminated model (III)

The interaction terms eliminated model was made using the same data set as the full model, using the fitlm function. Prior to making the model, the regression equation is declared in Wilkinson notation and then used as an argument in the function. Subsequently, the model was made, the p-values for each term assessed to be above 0.5 and then outliers identified in the same way as with the stepwise eliminated model. In this model the outliers were found to be run 7, 16, 20, 22, 28 and run 30. These were then excluded from the model. The data list for the PRESS was made using only the terms and response in the regression equation. Outliers were removed from this data list using a for-loop. Residual plots were made the same way. Prediction slice plots were made using the plotSlice function.

Table S13. Estimated Coefficients for interaction terms eliminated regression model and corresponding statistical values like the square error, t-statistic and the p-value.

	Estimate	SE	tStat	pValue
(Intercept)	16.705	3.40E+00	4.9143	0.000112
x1	-4936.9	382.09	-12.921	1.52E-10
x2	0.040648	0.0061137	6.65E+00	3.07E-06
x3	1.3553	1.60E-01	8.48	1.06E-07
x4	-2.1133	0.82233	-2.57E+00	1.93E-02
x4^2	-0.21516	0.057568	-3.74E+00	0.001507

Number of observations: 24, Error degrees of freedom: 18

Root Mean Squared Error: 0.413

R-squared: 0.979, Adjusted R-Squared 0.973

F-statistic vs. constant model: 165, p-value = 2.24e-14

PRESS: 6.1608, Predicted R-Squared: 0.9572

Table S14. Analysis of Variance (ANOVA) of the interaction terms eliminated regression model.

	SumSq	DF	MeanSq	F	pValue
Total	143.86	23	6.25E+00		
Model	140.78	5	2.82E+01	164.77	2.24E-14
. Linear	138.4	4	3.46E+01	202.47	1.06E-14
. Nonlinear	2.3871	1	2.3871	13.969	0.001507
Residual	3.0759	18	0.17088		
. Lack of fit	2.8527	13	0.21944	4.9159	0.044752
. Pure error	0.22319	5	0.044638		

Table S15. Analysis of Variance (ANOVA) of the terms of the interaction terms eliminated regression model.

	SumSq	DF	MeanSq	F	pValue
x1	28.529	1	2.85E+01	1.67E+02	1.52E-10
x2	7.5537	1	7.5537	4.42E+01	3.07E-06
x3	12.288	1	1.23E+01	7.19E+01	1.06E-07
x4	84.663	1	8.47E+01	495.45	1.51E-14
x4^2	2.3871	1	2.39E+00	13.969	0.001507
Error	3.0759	18	0.17088		

Table S16. Values corresponding to the goodness of fit (Figure 3c in manuscript) plot of the interaction terms eliminated regression model.

Run	yhat	y
1	-3.3286	-3.47557
2	-3.3286	-3.52813
3	-3.3286	-3.35256
4	-3.3286	-3.43822
5	-3.3286	-3.67822
6	-4.5705	-5.17236
8	-3.3834	-2.86742
9	-4.1416	-3.74557
10	-2.6917	-2.52842
11	-3.3286	-3.05006
12	-8.2174	-8.60154
13	-0.5709	-0.36515
14	-2.4497	-2.01946
15	-2.1968	-2.38431
17	-7.2837	-7.70377
18	-5.4048	-5.42405
19	-4.0756	-4.42348
21	-1.9661	-1.37117
23	-5.2330	-5.63214
24	-5.4859	-5.32472
25	-10.3199	-9.95072
26	-3.6071	-4.20106
27	-7.1118	-6.89505
28	-8.4410	-9.61217
29	-10.0669	-9.61217

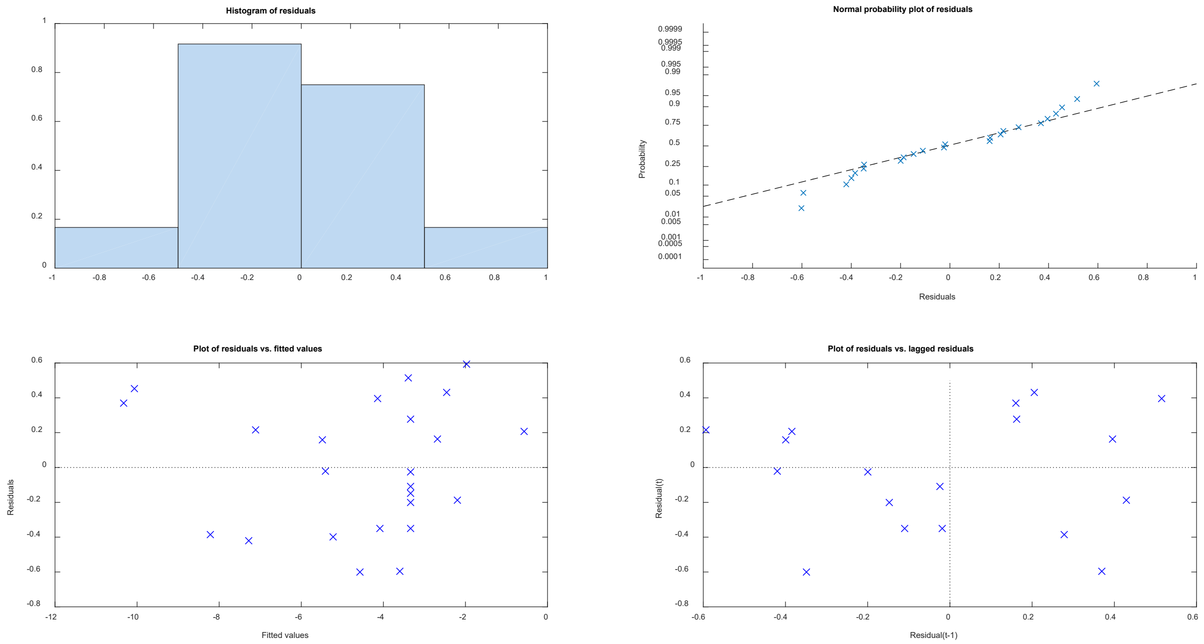

Figure S3. Assortment of residual plots corresponding to the interaction terms eliminated regression model. Top left: Histogram of residuals. Top right: Normal probability plot of residuals. Bottom left: residual plot. Bottom right: Lagged residual plot.

Figure S4. Prediction slice plots made using the interaction terms eliminated regression model. The interactive plot was made using plotSlice(mdl) and shows the predicted response for each variable as each

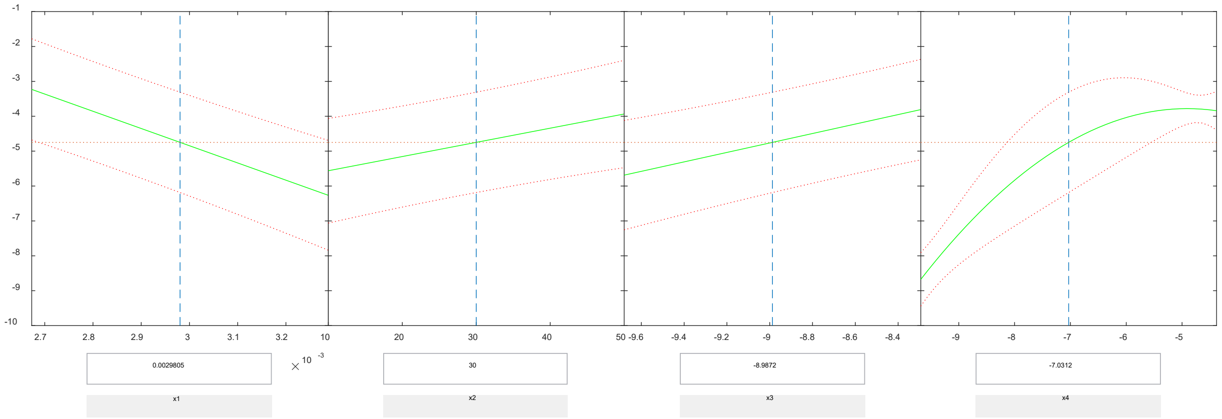

other variable is kept constant. 95% confidence bounds are also shown.

Stepwise eliminated model using TOF as a response

This model was made identically to the stepwise eliminated model, but with the TOF as a response. The turnover frequency (TOF) is a value obtained by dividing the hypothetical rate, which so far the natural log thereof was used as the response, by the concentration of the catalyst used for that run. No outliers were removed because the point of this exercise was to observe the p-values for each term. Due to the hypothetical reaction rate being normalised by the catalyst concentration, naturally the catalyst concentration variable was insignificant, and was not included in the model except as interaction term with the temperature. The point of this model was to show why the interaction terms could be just from overfitting.

Table S17. Analysis of Variance (ANOVA) of the terms of the stepwise eliminated regression model using TOF as a response.

	SumSq	DF	MeanSq	F	pValue
x1	45.123	1	45.123	96.601	1.06E-09
x2	7.5901	1	7.5901	16.249	0.00052081
x4	136.24	1	136.24	291.67	1.47E-14
x1:x3	0.34254	1	0.34254	0.73332	0.40064
x2:x4	1.714	1	1.714	3.6694	0.067935
x4^2	2.1966	1	2.1966	4.7025	0.040708
Error	10.743	23	0.4671		

Interaction term-eliminated model main effects

The z-transformed main effects were obtained in the same way as with the stepwise eliminated model.

Table S18. Main effect sizes as estimated coefficients from an interaction terms eliminated regression model built from z-transformed variables and response.

	effects
Pressure	0.19427
Catalyst	0.26223
Base ^2	0.35268
Temperature	0.49545
Base	0.50023

The DoE normalised main effects were obtained in the same way as with the stepwise eliminated model.

Table S19. Main effect sizes as T-statistic from an interaction terms eliminated regression model built from DoE normalised variables.

	T-statistic
pressure	4.7706
catalyst	6.2905
temperature	11.661
base ^2	12.794
base	16.669

4.4. Nonlinear regression model (IV)

The response values are the same as for the other models. The model was made using the function “fitnlm” with the variables, response, regression equation (in Wilkinson notation), initial coefficient estimates and options for robust fitting. The regression equation used was 'y ~ b7 - b1 * (x1/0.008314) + b2 * x2 + b3 * x3 + b4 * x4 + x5 + b6 * x6' (with b7 being the intercept). The initial coefficient estimates was a 6 by 1 matrix of ones. These would be used by the fitting algorithm to start estimating what the coefficients will be. For the robust fitting a structure variable of options have to be created. This is done by using the function “statset” with ‘nlinfit’ as argument. Then the parameter “RobustWgtFun” was set to 'bisquare', which is the equation used for the weighting. The parameter “MaxIter” was set to 9999999999999. This was the maximum amount of iterations the program would do to find the coefficients. After the model was made, outliers were identified by finding the standardized residuals above 1.25 and below -1.25 and subsequently removed. These runs were removed from the data set for the PRESS as well. The outlier runs were run 7, 10, 22, 25, 28 and run 30. The variance inflation factor was calculated using first the function “corrcoef” as such “R0 = corrcoef(X)” with the variables being X. Subsequently the VIF’s are obtained with this code “VIF = diag(inv(R0))”.

Table S20. Estimated Coefficients for nonlinear regression model and corresponding statistical values like the square error, t-statistic and the p-value.

	Estimate	SE	tStat	pValue
Intercept	31.554	4.0357	7.8187	3.39E-07
x_1	39.307	7.5597	5.1995	6.04E-05
x_2	0.035241	0.0092655	3.8035	0.0013012
x_3	1.5982	0.27672	5.7755	1.79E-05
x_4	1.0932	0.07059	15.487	7.54E-12
x_6	-3.70E-05	8.84E-06	-4.19E+00	0.00055007

Number of observations: 24, Error degrees of freedom: 18

Root Mean Squared Error: 0.657

R-Squared: 0.955, Adjusted R-Squared 0.942

F-statistic vs. constant model: 75.6, p-value = 1.93e-11

PRESS: 10.2716, Predicted R-Squared: 0.9221

Table S21. Values corresponding to the goodness of fit plot (Figure 3d in manuscript) of the nonlinear regression model.

Run	y	y_hat
1	-3.47557	-3.159330
2	-3.52813	-3.168343
3	-3.35256	-3.277272
4	-3.43822	-3.216649
5	-3.67822	-3.080277
6	-5.17236	-4.253648
8	-2.86742	-3.176041
9	-3.74557	-3.765227
11	-3.05006	-3.594488
12	-8.60154	-7.758773
13	-0.36515	0.066503
14	-2.01946	-2.552334
15	-2.38431	-2.413034
16	-5.81914	-6.845135
17	-7.70377	-7.614007
18	-5.42405	-5.453929
19	-4.42348	-3.994940
20	-9.73691	-9.545654
21	-1.37117	-2.593179
23	-5.63214	-5.244720
24	-5.32472	-6.080677
26	-4.20106	-4.155006
27	-6.89505	-7.399304
29	-9.61217	-9.890377

Leave-one-out-cross-validation.

The LOOCV methodology iteratively removes one run, makes the model and uses the corresponding estimated coefficients to provide a prediction for that removed run. The predicted response can then be compared with the actual response.

Table S22. Values corresponding to the LOOCV goodness of fit plot (Figure 5 in manuscript) of the nonlinear regression model .

Run	pred hyprate	LOOCV result
1	-3.159330	-3.138005
2	-3.168343	-3.144287
3	-3.277272	-3.272227
4	-3.216649	-3.201055
5	-3.080277	-3.043492
6	-4.253648	-4.018281
8	-3.176041	-3.201576
9	-3.765227	-3.768979
11	-3.594488	-3.636703
12	-7.758773	-7.510371
13	0.066503	0.315458
14	-2.552334	-2.831904
15	-2.413034	-2.427710
16	-6.845135	-7.277614
17	-7.614007	-7.554921
18	-5.453929	-5.499849
19	-3.994940	-3.644555
20	-9.545654	-9.417746
21	-2.593179	-2.860434
23	-5.244720	-5.056489
24	-6.080677	-6.552110
26	-4.155006	-4.113711
27	-7.399304	-7.680155
29	-9.890377	-10.302524

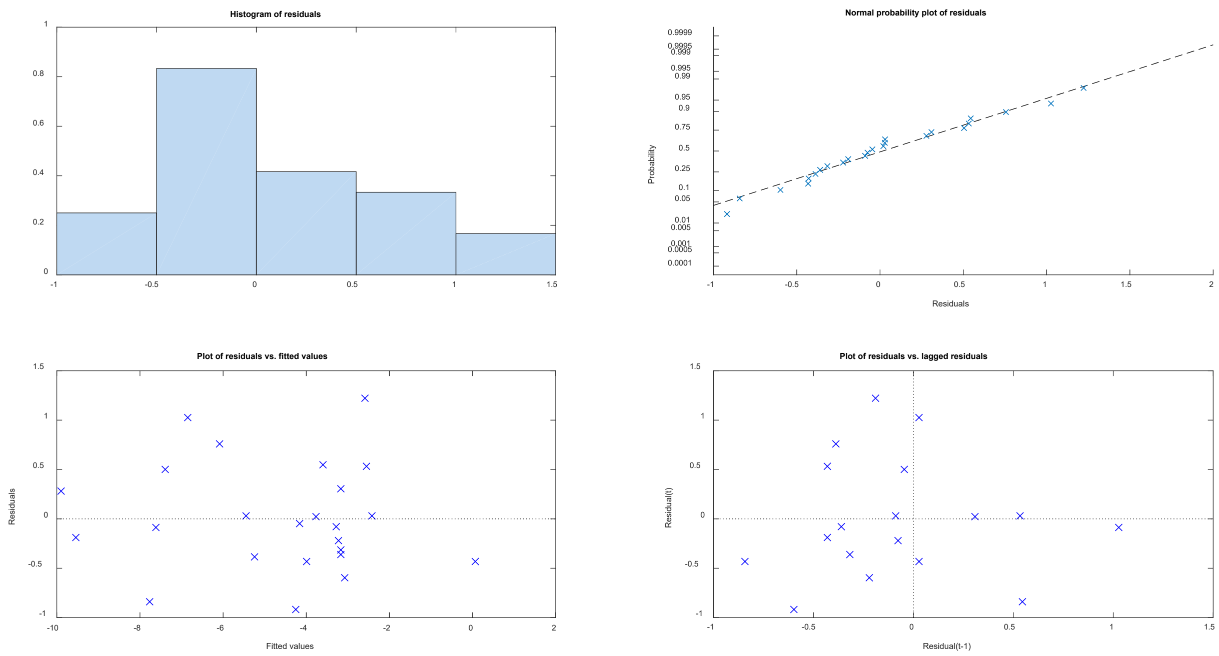

Figure S5. Assortment of residual plots corresponding to the nonlinear regression model. Top left: Histogram of residuals. Top right: Normal probability plot of residuals. Bottom left: residual plot. Bottom right: Lagged residual plot.

5. Catalytic results

Table S23. Values corresponding to the 50 ppm catalyst concentration Arrhenius plot. Multiple experiments were performed at 85˚C to indicate reproducibility. Slope (Figure 7A in manuscript) roughly corresponds to ~90 KJ/mol.

Inverse of temperature (1/K)	Ln(k)
0.00319	-16.81304
0.00279	-12.10575
0.00279	-11.87639
0.00279	-11.57612
0.00279	-11.56225
0.00279	-11.80209
0.00279	-11.47901
0.00248	-9.21688

Table S24. Values corresponding to the 200 ppm catalyst concentration Arrhenius plot. Slope (Figure 7A in manuscript) roughly corresponds to 51 KJ/mol.

Inverse of temperature (1/K)	Ln(k)
0.0033	-11.76008
0.00314	-11.25687
0.003	-10.06535
0.003	-9.70741
0.00291	-9.59154
0.00283	-8.69802
0.00268	-8.39501
0.00268	-7.88619

Table S25. Values corresponding to the log plot of reaction rates vs base concentration. Two linear regions were fitted, for which the first has its equation displayed.

Ln(base PPM)	Ln(reaction rate)
4.60517	-15.46555
5.29832	-13.7406
5.70378	-12.77638
5.99146	-11.98683
8.51719	-10.33027
9.21034	-10.43471
9.21034	-10.40126
9.90349	-10.35417
10.81978	-10.2148

Table S26. Values corresponding to the log plot of reaction rates vs catalyst concentration. Last data point was not included in the fitting.

Ln(cat PPM)	Ln(reaction rate)
0.69315	-16.14937
1.60944	-14.66447
2.30259	-14.06687
3.21888	-12.42802
3.91202	-11.30685
4.60517	-9.99534
5.29832	-10.06535

6. References

[1] W. Yang, I. Y. Chernyshov, R. K. van Schendel, M. Weber, C. Müller, G. A. Filonenko, E. A. Pidko, *Nat. Commun.* **2021**, *12*, 1-8.

[2] Antonio Trujillo-Ortiz (2021). press (https://www.mathworks.com/matlabcentral/fileexchange/14564-press), MATLAB Central File Exchange. Retrieved July 28, 2021.
